# Vitamin A prevents lipopolysaccharide‐induced injury on tight junctions in mice

**DOI:** 10.1002/fsn3.1481

**Published:** 2020-02-27

**Authors:** Caimei He, Xin Hu, Di Xiao, Jingtao Wu, Sichun Zhou, Jun Deng, Simeng Xu, Yanjun Huang, Mei Peng, Xiaoping Yang

**Affiliations:** ^1^ Key Laboratory of Study and Discovery of Small Targeted Molecules of Hunan Province Department of Pharmacy School of Medicine Hunan Normal University Changsha China; ^2^ Department of Pharmacy Traditional Chinese Hospital of Yueyang City Yueyang China; ^3^ Department of Pharmacy Xiangya Hospital Central South University Changsha China

**Keywords:** inflammation, intestine, tight junctions, vitamin A

## Abstract

Vitamin A (VA) is one of the most widely used food supplements, but its molecular mechanisms largely remain elusive. Previously, we have demonstrated that VA inhibits the action of lipopolysaccharide (LPS) on intestinal epithelial barrier function and tight junction proteins using IPEC‐J2 cells, one of representative intestinal cell lines as a cellular model. These exciting findings stimulated us continue to determine the effects of VA on LPS‐induced damage of intestinal integrity in mice. Our results demonstrated that LPS treatment caused reductions of the mRNA levels of tight junction proteins including Zo‐1, Occludin, and Claudin‐1, well‐known biomarkers of intestinal integrity, and these reductions were reversed by VA pretreatment. Intestinal immunofluorescent results of Claudin‐1 revealed that LPS disrupted the structure of tight junction and reduced the expression of Claudin‐1 at protein level, which was reversed by VA pretreatment. These results suggest that VA may exert a profound role on preventing intestinal inflammation in vivo.

## Graphical abstract

Vitamin A (VA) is one of the most widely used food supplements, but its molecular mechanisms largely remain elusive. These results suggest that VA may exert a profound role on preventing intestinal inflammation in vivo and correcting LPS-induced disruption of tight junction proteins.

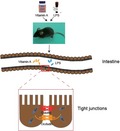

## 1 INTRODUCTION

Vitamin A (VA) is a commonly used food supplement that maintains visual function, tissue development, differentiation, and immune response. Particularly, VA has been confirmed having its critical role of anti‐inflammatory effect (Xiao et al., 2019; Zhang et al., 2019). Interestingly, VA plays an essential role in tight junctions in vitro (Osanai, 2017; Rybakovsky et al., 2017). Tight junctions are important components of the intestinal epithelial barrier. The development of most diseases is related to the intestinal epithelial barrier in the clinic, and the intestine has a function of separating the substances in the intestinal lumen and preventing the invasion of pathogenic antigens (Buckley & Turner, 2018; De Santis, Cavalcanti, Mastronardi, Jirillo, & Chieppa, 2015). Thus, alteration of tight junctions is becoming an important biomarker of determining the function of intestine (Wardill, Gibson, Logan, & Bowen, 2014). Studies have confirmed that LPS, one commonly and important inflammation activator, produces damages on tight junctions in vitro (Chen et al., 2015; Main, Weber, Baumgard, & Gabler, 2012). Previously, we have shown that VA protected against LPS‐induced damage on tight junctions in vitro (He et al., 2019). However, whether or how effect of VA on tight junction of intestine in vivo remains elusive. Thus, we use miniature mice to further explore the effect of VA on tight junctions in vivo.

## 2 MATERIALS AND METHODS

### 2.1 Materials

Vitamin A was purchased from Sigma‐Aldrich and dissolved in DMSO to prepare a stock solution of 100 mM. LPS was purchased from Sigma‐Aldrich as well and dissolved in PBS to prepare the solution stored at −20°C. IL‐6, TNF‐α, and β‐actin were purchased from Cell Signaling Technology. Sodium carboxymethyl cellulose was purchased from Aladdin Biochemical Technology Co., Ltd.

### 2.2 Animals and treatments

Specific pathogen‐free male C57BL/6N mice weighing 20–22 g (purchased from Hunan Slack Jing da Experimental Animal Co., Ltd) were housed in specific pathogen‐free conditions at Hunan Normal University, China. Animal experiments were performed in accordance with the National Guideline for Experimental Animal Welfare and with approval of the Animal Welfare and Research Ethics Committee at Hunan Normal University (approved animal protocol number 201808, extended animal protocol number 2019041). C57BL/6N mice were randomly divided into four groups: control, LPS (5 mg/kg), VA (1 mg/kg), LPS (5 mg/kg) + VA (1 mg/kg) groups. The mice were treated with oral VA for 4 days and intraperitoneal LPS for 24 hr. The doses of LPS and VA were adopted from previous published work (Kim, Kim, Park, Kim, & Chang, 2015).

### 2.3 Collection of mice intestinal tissue samples

At the end of each experiment, the mouse cervical spine was dislocated and the intestinal tissues were collected immediately. After washing with PBS at 4°C, and intestinal tissues were either for cryosections or stored in liquid nitrogen for further study.

### 2.4 Reverse transcriptase‐polymerase chain reaction

The PCR system consisted of 5.0 μl of YBR Green qPCR Mix, 0.2 μl of cDNA, 0.3 μl of each primer, and 4.2 μl of double‐distilled water in a final volume of 20 μl. The detailed information of each primer was presented in Table 1. Each sample was determined in triplicate, and the housekeeping gene GAPDH was used as the internal standard for the PCR. Quantitative real‐time PCR was performed with a Real Master Mix SYBP ROX (5 Prime) according to the manufacturer's protocols using the same pig GAPDH primer.

**Table 1:** The detailed information of primers for RT‐PCR

Gene	Forward primer (5′–3′)	Reverse primer (5′–3′)
GAPDH	GAAGGTCGGAGTGAACGGAT	CTGGCATTGACTGGGGTCA
Zo‐1	AGCTGCCTCGAACCTCTACTCTAC	GCCTGGTGGTGGAACTTGCTC
Occludin	TGGCTATGGAGGCGGCTATGG	AAGGAAGCGATGAAGCAGAAGGC
Claudin‐1	GGTGCCTGGAAGATGATGAGGTG	GCCACTAATGTCGCCAGACCTG
TNF‐α	GCGACGTGGAACTGGCAGAAG	GCCACAAGCAGGAATGAGAAGAGG
IL‐6	ACTTCCATCCAGTTGCCTTCTTGG	TTAAGCCTCCGACTTGTGAAGTGG

### 2.5 Western blot

Tissues proteins were fractionated by sodium dodecyl sulfate–polyacrylamide gel electrophoresis, transferred to membranes, and then incubated overnight at 4°C with different primary antibodies described in Reagents section above (Cell Signaling Technology) in buffer containing bovine serum albumin. Membranes were washed with triethanolamine‐buffered saline (TBS) containing 0.05% Tween‐20, blotted with secondary antibody for 1 hr at room temperature, and then washed again three times. Pierce Super Signal chemiluminescent substrate (Rockford, IL, USA) was added, and the blot was imaged immediately on a ChemiDoc system (Tanon 4600) and a Perfection V500 camera (Epson). Band intensities were quantified using ImageJ.

### 2.6 Preparation of cryosections

Each intestine tissue was transferred from fixative to 300 μl 2.3 M sucrose in 0.1 M sodium phosphate pH 7.4 at 4°C for 1 hr, then embedded and quick‐frozen in OCT compound (Tissue‐Tek). 5‐μm cryosections were cut at −20°C collected on chrome alum/gelatin‐subbed slides, and stored up to 24 hr at −20°C. Optimally oriented cryosections were selected for further processing.

### 2.7 Immunofluorescence microscopy

Localization of Claudin‐1, one representative of tight junction proteins in intestine in each group, was determined by immunofluorescence microscopy. First, cryosection of intestine tissues was washed with PBS. For Claudin‐1 staining, cryosection of intestine tissues was fixed with 2% (v/v) formaldehyde in PBS for 30 min and incubated with 1% (v/v) Triton X‐100 in PBS 3 times for 5 min each to permeabilize cells and then washed and blocked for 30 min with 1% (w/v) bovine serum albumin (BSA) in PBS. Cryosection was then incubated with primary antibody against rabbit Claudin‐1 (1:100) overnight at 4°C. Cryosection was then washed again with PBS and incubated with the secondary antibody, Alexa Fluor 488 goat anti‐rabbit (1:100) (Proteintech). Cryosection was then washed again with PBS, and the permeable support membrane was then mounted side up between a slide and coverslip with a DAPI‐containing mounting medium (Beyotime Biotechnology). Microscopic images of the mounted membranes were taken on a Zeiss LSM 510 Meta Confocal Laser Scanning Microscope (Leica, DFC450C). Scan areas were chosen based on intact tissue structures, avoiding the edges of the square cutouts.

### 2.8 Statistical analysis

SPSS16.0 (SPSS Inc.) was used for statistical analysis. Measurement data were presented as mean ± standard deviation (*SD*). Comparison of means among multiple groups was performed by one‐way analysis of variance (ANOVA), followed Dunnett post hoc test. A statistical significance was defined when *p* < .05.

## 3 RESULTS

### 3.1 Effect of vitamin A on gene expression of tight junction in intestinal tissues in mice

**Figure 1:**
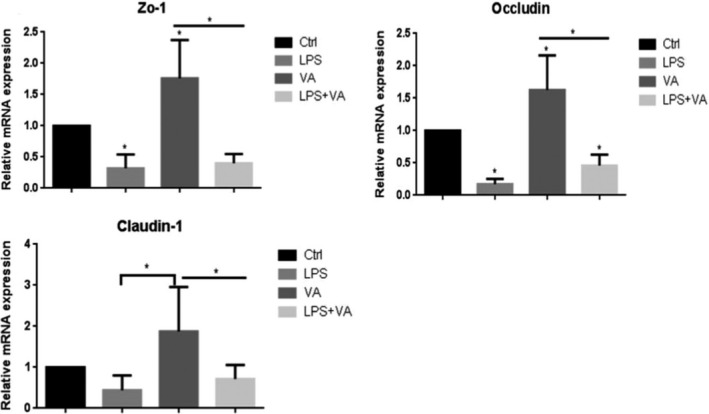
RT‐PCR results of Zo‐1, Occludin, and Claudin‐1 in intestine tissues from control, LPS (5 mg/kg), VA (1 mg/kg), LPS (5 mg/kg) + VA (1 mg/kg) groups. Treatment with 5 mg/kg LPS significantly decreases mRNA levels of Zo‐1, Occludin, and Claudin‐1, indicating damage of epithelial integrity. In contrast, 1 mg/kg VA enhances mRNA expression of tight junction (*p < .05, compared with control, *n* = 4 for each group)

We utilized intestine tissues among four groups to determine whether there is any alteration of tight junctions. mRNA levels of Zo‐1, Occludin, and Claudin‐1 were determined by Reverse transcriptase‐polymerase chain reaction (RT‐PCR). As shown in Figure 1, VA increased gene expression of Zo‐1, Occludin, and Claudin‐1. In contrast, as we expected, LPS downregulated Zo‐1, Occludin, and Claudin‐1 at mRNA Levels. Interestingly, mRNA levels of Zo‐1, Occludin, and Claudin‐1 with cotreatment of VA and LPS were significantly increased compared with the treatment of LPS alone. These observations clearly demonstrated that treatment of VA attenuated LPS‐induced decrease in these tight junctions at mRNA levels, indicating that VA inhibits the action of LPS on tight junctions in vivo.

### 3.2 Effect of Vitamin A on inflammatory responses in mice

**Figure 2:**
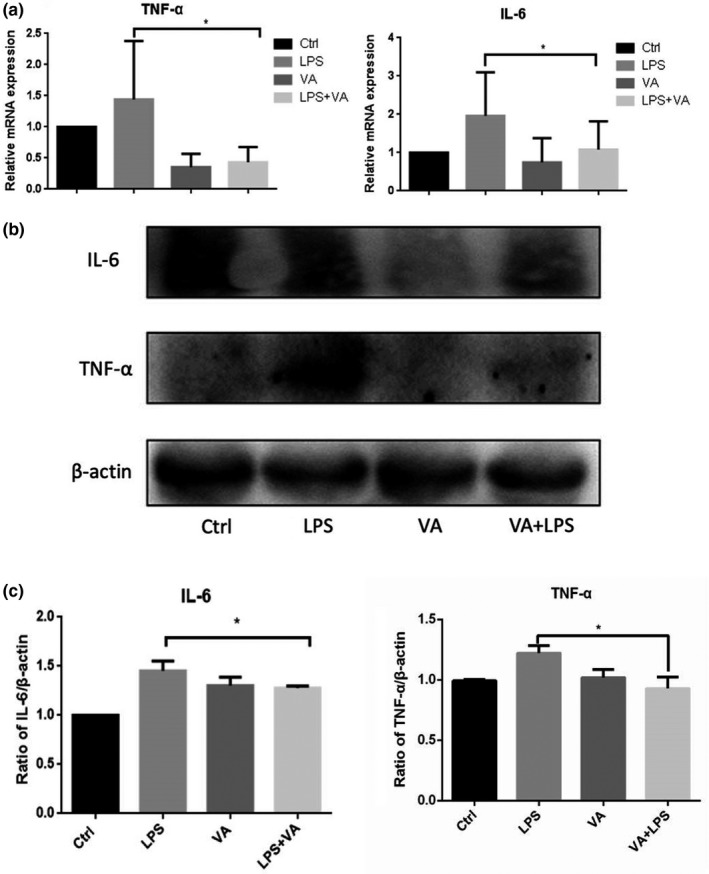
Relative mRNA expression of TNF‐α and IL‐6 from intestine tissues of mice after the treatment of control, LPS (5 mg/kg), VA (1 mg/kg), LPS (5 mg/kg) + VA (1 mg/kg) (*p < .05, compared with control, *n* = 4 for each group) (a); WB results of TNF‐α and IL‐6 proteins from intestine tissues of mice after the treatment of control, LPS (5 mg/kg), VA (1 mg/kg), LPS (5 mg/kg) + VA (1 mg/kg) (*p < .05, compared with control, *n* = 4 for each group) (b); statistics results of WB results (c). VA dramatically alleviated LPS‐induced increases of TNF‐α and IL‐6 at both mRNA and protein levels

To explore anti‐inflammatory function of VA on intestinal tissues, alterations of TNF‐α and IL‐6 as biomarkers of inflammation during these treatments were examined. As shown in Figure 2a, LPS increased TNF‐α and IL‐6 expressions, while VA alone significantly decreased TNF‐α and IL‐6 expression at mRNA levels. Interestingly, cotreatment of VA and LPS exhibited profoundly attenuated effect of VA on LPS‐induced inflammation, and evidenced by significant decrease in TNF‐α and IL‐6 compared with LPS treatment group. Profoundly, similar trend at protein levels was observed via Western Blot (Figure 2b,c).

### 3.3 Immunofluorescent evaluation of the localization and expression of tight junction

**Figure 3:**
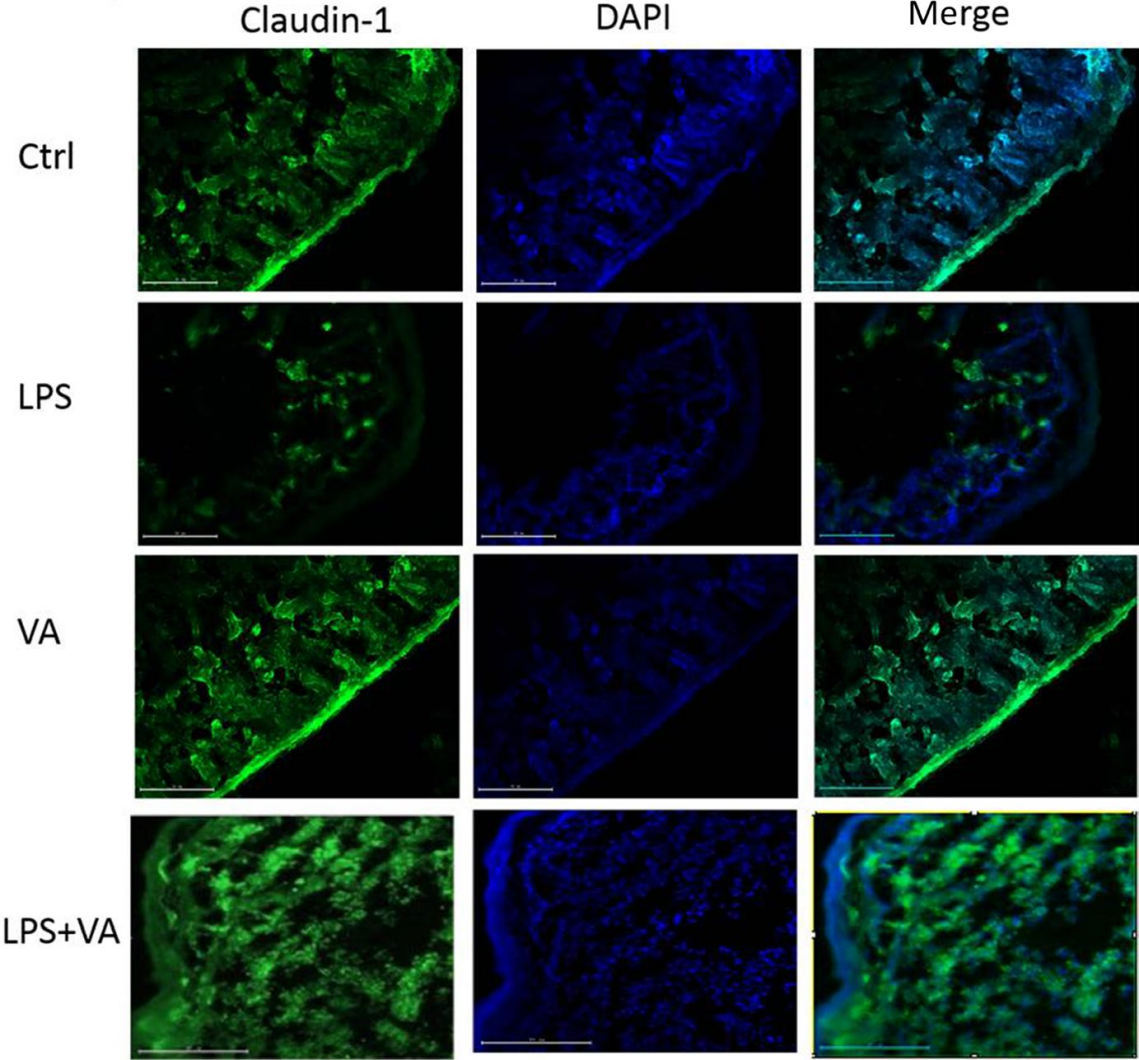
Representative immunofluorescent images of cryosections of intestine tissues from the treatments of control, LPS (5 mg/kg), VA (1 mg/kg), LPS (5 mg/kg) + VA (1 mg/kg) (*n* = 4 for each group). Primary antibody Claudin‐1 was used as one of biomarkers of tight junction proteins, while DAPI was used for counter‐staining nucleus. LPS caused obviously severe disruption of Claudin‐1 structurally, while VA dramatically prevented this disruption. Consistent observations regarding protein expressions of Claudin‐1 were collected as well

Immunofluorescence was used to detect the localization and expression of tight junctions since results collected by immunofluorescence microscopy are more intuitive. As shown in Figure 3, tight junction of Claudin‐1 is neatly arranged in mouse intestine tissues in the control group, while VA alone profoundly enhanced Claudin‐1 expression with perfect arrangement. However, LPS‐treated group showed severely structural disruption with decrease in tight junction protein Claudin‐1. In contrast, VA treatment protected this disruption when the mice were treated with both VA and LPS.

## 4 DISCUSSION

Vitamin A is one of the most conventional daily food supplements with minimum toxicity and great nutritional values. It new functions have continuously attracted scientific attentions. Okayasu et al. (2016) disclosed that VA can block development of dextran sulfate sodium‐induced colitis and colon cancer using a mouse model. Holloway, Kim, and Quadro (2019) disclosed that the homeostasis RA, the active form of VA, may be involved in modulating the cardiac hypertrophy of pregnancy and VA can enhance cardiac function. Applying VA deficiency model, Qi et al. (2019) explored novel changes of genes involved in nutrition metabolism and immune responses in growth retardation piglets. Cui et al. (2019) explored that VA deficiency enhances development of Lewis lung carcinoma through induction of type 2 innate lymphoid cells and activates macrophages. Thus, supplying VA could attenuate development of Lewis lung carcinoma by improving type 2 immune response (Cui et al., 2019).

These important findings mentioned above imply that VA has very likely potential function on strengthening immune system. In contrast, intestinal inflammation induced by toxins is a deadly clinical problem. Preventing this intestinal inflammation problem has an unmet clinical demand. LPS, one of the important immune modulators, is widely used as an efficient model for mimicking inflammation event induced by toxins.

Recently, it has been reported that the development of inflammation ‐caused diseases is associated with alteration of intestinal tissue proteins, particularly tight junction proteins (Yang et al., 2019). Our previous study has shown that VA inhibits the action of LPS on the intestinal epithelial barrier function and tight junction proteins at cellular level (He et al., 2019). However, it is unknown whether this protective effect of VA could be seen in vivo or not. Thus, in this study we continue to explore whether this inhibitory effect of VA exists in vivo or not. Our data show that VA alone has dramatic effect on increasing the expression of tight junctions both at RNA levels (RT‐PCR) and protein levels (IF). Combined them together, VA could attenuate LPS‐boosted increase in inflammation biomarkers and reverse the LPS‐caused decrease in tight junction proteins simultaneously. Furthermore, VA can prevent LPS‐induced disruption and reduction in tight junctions. We are the first to reveal the protective effect of VA on LPS‐induced disruption of tight junctions and to clearly observe the location of tight junctions in mouse intestines.

**Figure 4:**
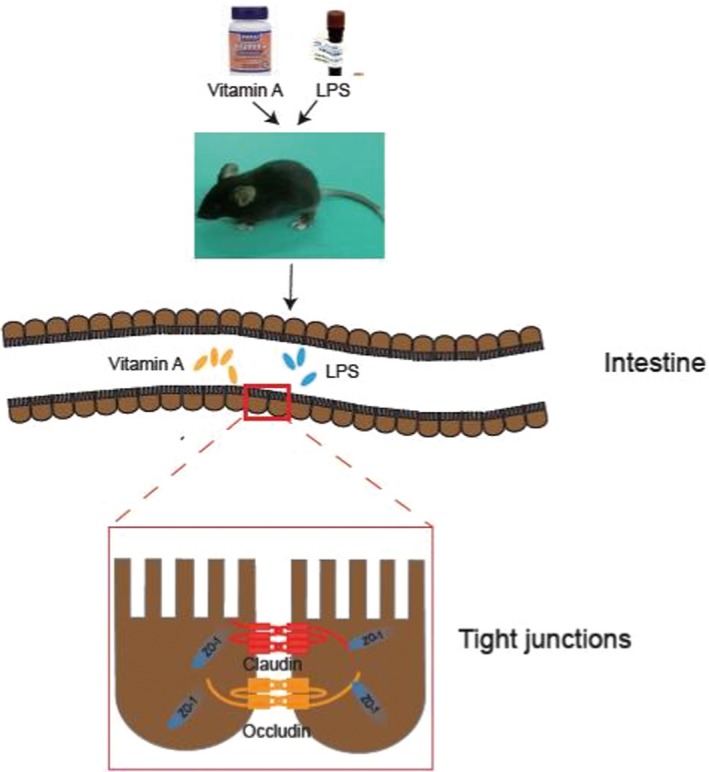
Working model describes protective effect of VA on LPS‐induced damage on intestines in mice. VA enhances expressions of tight junctions, while LPS decreases their expressions. Pretreatment of VA shows a profound effect on enhancing LPS‐induced decrease in intestinal tight junctions and correcting LPS‐caused disruptive alteration of intestinal tight junctions in mice

Recently, Fan, Liu, Song, Chen, and Ma (2017) found that moderate dietary protein restriction could promote colonization of beneficial bacteria in both ileum and colon, then enhance tight junction protein function, indicating the importance of tight junction protein. Moreover, sodium butyrate reduced antibiotics in a piglet diet in promoting performance and to control weaning diarrhea by the modulation of intestinal permeability and the bacterial communities in the ileum and colon (Huang et al., 2015). Interestingly, Sterlin et al. (2019) reported that human gut microbiomes evolved in the absence of immunoglobulin A (lgA). Furthermore, Ma et al. (2018) summarized that intestinal bacteria–immune crosstalk and nutritional regulation on their interplay, to understanding their interactions. In contrast, another report does not support that a short‐term pro‐inflammatory effect of *A. muciniphila* strain in the IL‐10^−/−^ mouse model for IBD (Ring et al., 2019). Another study reveals that RegIIIb‐target recognition, killing of Gram‐negative bacteria in infectious diarrhea, proposes avenues toward novel therapeutic interventions for Salmonella diarrhea (Miki, Okada, & Hardt, 2018). Using human patient specimens, Kiely, Pavli, and O'Brien (2018) demonstrated that inflammation is related to microbiome, which is spatially and timely altered in inflammatory bowel disease (IBD). Interestingly, Mamantopoulos, Ronchi, McCoy, and Wullaert (2018) analyzed the association between maternal inheritance and long‐term separate housing and host–microbiota interactions, illustrating the importance of host–microbiota and inflammatory responses. As summarized in Figure 4, we find that VA enhances expressions of tight junctions, while LPS decreases their expressions in mice, consistent with our previous observation in vitro. Pretreatment of VA shows a profound effect on enhancing LPS‐induced decrease in intestinal tight junctions and correcting LPS‐caused disruptive alteration of intestinal tight junctions in mice. Dramatically, intestinal immunofluorescent results of Claudin‐1 revealed that LPS disrupted the structure of tight junction and reduced the expression of Claudin‐1, while VA pretreatment reversed the two events.

## 5 CONCLUSIONS

The present study reveals that VA enhances expression of intestinal tight junction proteins and reverses both LPS‐induced increase in inflammation and decrease in intestinal tight junction proteins. These results for the first time confirm that VA plays a profound role on preventing intestinal inflammation.

## CONFLICT OF INTEREST

The authors declare that they have no competing interests.

## ETHICAL APPROVAL

Animal experiments were performed in accordance with the National Guideline for Experimental Animal Welfare and with approval of the Animal Welfare and Research Ethics Committee at Hunan Normal University (approved animal protocol number 201808, extended animal protocol number 2019041).

## References

[fsn31481-bib-0001] Buckley, A. , & Turner, J. R. (2018). Cell Biology of tight junction barrier regulation and mucosal disease. Cold Spring Harbor Perspectives in Biology, 10 pii:a029314. 10.1101/cshperspect.a029314 PMC5749156 28507021

[fsn31481-bib-0002] Chen, J. , Zhang, R. , Wang, J. , Yu, P. , Liu, Q. , Zeng, D. , … Kuang, Z. (2015). Protective effects of baicalin on LPS‐induced injury in intestinal epithelial cells and intercellular tight junctions. Canadian Journal of Physiology and Pharmacology, 93, 233–237. 10.1139/cjpp-2014-0262 25665915

[fsn31481-bib-0003] Cui, W. , Zhang, W. , Yuan, X. , Liu, S. , Li, M. , Niu, J. , … Li, D. (2019). Vitamin A deficiency execrates Lewis lung carcinoma via induction of type 2 innate lymphoid cells and alternatively activates macrophages. Food Sciences and Nutrition, 7, 1288–1294. 10.1002/fsn3.961 PMC6475724 31024701

[fsn31481-bib-0004] De Santis, S. , Cavalcanti, E. , Mastronardi, M. , Jirillo, E. , & Chieppa, M. (2015). Nutritional keys for intestinal barrier modulation. Frontiers in Immunology, 6, 612 10.3389/fimmu.2015.00612 26697008 PMC4670985

[fsn31481-bib-0005] Fan, P. , Liu, P. , Song, P. , Chen, X. , & Ma, X. I. (2017). Moderate dietary protein restriction alters the composition of gut microbiota and improves ileal barrier function in adult pig model. Scientific Reports, 7, 43412 10.1038/srep43412 28252026 PMC5333114

[fsn31481-bib-0006] He, C. , Deng, J. , Hu, X. , Zhou, S. , Wu, J. , Xiao, D. , … & Yang, X. (2019). Vitamin A inhibits the action of LPS on the intestinal epithelial barrier function and tight junction proteins. Food & function, 10(2), 1235–1242. 10.1039/c8fo01123k 30747184

[fsn31481-bib-0007] Holloway, C. , Kim, Y. K. , & Quadro, L. (2019). The role of beta‐carotene metabolism in maternal cardiac remodeling: Findings in mice lacking beta‐carotene ,10‐oxygenase (BCO2) (FS06‐04‐19). Current Developments in Nutrition, 3.

[fsn31481-bib-0008] Huang, C. , Song, P. , Fan, P. , Hou, C. , Thacker, P. , & Ma, X. I. (2015). Dietary sodium butyrate decreases postweaning diarrhea by modulating intestinal permeability and changing the bacterial communities in weaned piglets. The Journal of Nutrition, 145(12), 2774–2780. 10.3945/jn.115.217406 26491121

[fsn31481-bib-0009] Kiely, C. J. , Pavli, P. , & O'Brien, C. L. (2018). The role of inflammation in temporal shifts in the inflammatory bowel disease mucosal microbiome. Gut Microbes, 9, 477–485. 10.1080/19490976.2018.1448742 29543557 PMC6287691

[fsn31481-bib-0010] Kim, Y. M. , Kim, J. H. , Park, S. W. , Kim, H. J. , & Chang, K. C. (2015). Retinoic acid inhibits tissue factor and HMGB1 via modulation of AMPK activity in TNF‐α activated endothelial cells and LPS‐injected mice. Atherosclerosis, 241(2), 615–623. 10.1016/j.atherosclerosis.2015.06.016 26116962

[fsn31481-bib-0011] Ma, N. , Guo, P. , Zhang, J. , He, T. , Kim, S. W. , Zhang, G. , & Ma, X. (2018). Nutrients mediate intestinal bacteria‐mucosal immune crosstalk. Frontiers in Immunology, 9, 5 10.3389/fimmu.2018.00005 29416535 PMC5787545

[fsn31481-bib-0012] Main, V. , Weber, T. E. , Baumgard, L. H. , & Gabler, N. K. (2012). Growth and Development Symposium: Endotoxin, inflammation, and intestinal function in livestock. Journal of Animal Science, 90, 1452–1465. 10.2527/jas.2011-4627 22247110

[fsn31481-bib-0013] Mamantopoulos, M. , Ronchi, F. , McCoy, K. D. , Wullaert, A. (2018). Inflammasomes make the case for littermate‐controlled experimental design in studying host‐microbiota interactions. Gut Microbes, 9(4), 374–381. 10.1080/19490976.2017.1421888 29672197 PMC6219641

[fsn31481-bib-0014] Miki, T. , Okada, N. , & Hardt, W. D. (2018). Inflammatory bactericidal lectin RegIIIβ: Friend or foe for the host? Gut Microbes, 9(2), 179–187. 10.1080/19490976.2017.1387344 28985140 PMC5989794

[fsn31481-bib-0015] Okayasu, I. , Hana, K. , Nemoto, N. , Yoshida, T. , Saegusa, M. , Yokota‐Nakatsuma, A. , … Iwata, M. (2016). Vitamin A inhibits development of dextran sulfate sodium‐induced colitis and colon cancer in a mouse model. BioMed Research International, 2016, 1–11. 10.1155/2016/4874809 PMC4889797 27298823

[fsn31481-bib-0016] Osanai, M. (2017). Cellular retinoic acid bioavailability in various pathologies and its therapeutic implication. Pathology International, 67, 281–291. 10.1111/pin.12532 28422378

[fsn31481-bib-0017] Qi, M. , Tan, B. E. , Wang, J. , Li, J. , Liao, S. M. , Yan, J. M. , … Yin, Y. (2019). Small intestinal transcriptome analysis revealed changes of genes involved in nutrition metabolism and immune responses in growth retardation piglets. Journal of Animal Science, 97, 3795–3808. 10.1093/jas/skz205 31231776 PMC6735748

[fsn31481-bib-0018] Ring, C. , Klopfleisch, R. , Dahlke, K. , Basic, M. , Bleich, A. , Blaut, M. (2019). Akkermansia muciniphila strain ATCC BAA‐835 does not promote short‐term intestinal inflammation in gnotobiotic interleukin‐10‐deficient mice. Gut Microbes, 10, 188–203. 10.1080/19490976.2018.1511663 30252588 PMC6546315

[fsn31481-bib-0019] Rybakovsky, E. , Valenzano, M. C. , Deis, R. , DiGuilio, K. M. , Thomas, S. , & Mullin, J. M. (2017). Improvement of human‐oral‐epithelial‐barrier function and of tight junctions by micronutrients. Journal of Agriculture and Food Chemistry, 65, 10950–10958. 10.1021/acs.jafc.7b04203 29172516

[fsn31481-bib-0020] Sterlin, D. , Fieschi, C. , Malphettes, M. , Larsen, M. , Gorochov, G. , & Fadlallah, J. (2019). Immune/microbial interface perturbation in human IgA deficiency. Gut Microbes, 10(3), 429–433. 10.1080/19490976.2018.1546520 30449244 PMC6546332

[fsn31481-bib-0021] Wardill, H. R. , Gibson, R. J. , Logan, R. M. , & Bowen, J. M. (2014). Tlr4/PKC‐mediated tight junction modulation: A clinical marker of chemotherapy‐induced gut toxicity? International Journal of Cancer, 135, 2483–2492. 10.1002/ijc.28656 24310924

[fsn31481-bib-0022] Xiao, L. , Chen, B. , Feng, D. , Yang, T. , Li, T. , & Chen, J. (2019). TLR4 may be involved in the regulation of colonic 2 mucosal microbiota by vitamin A. Frontiers in Microbiology, 10, 268 10.3389/fmicb.2019.00268 30873131 PMC6401601

[fsn31481-bib-0023] Yang, H. S. , Haj, F. G. , Lee, M. , Kang, I. , Zhang, G. , & Lee, Y. (2019). *Laminaria japonica* extract enhances intestinal barrier function by altering inflammatory response and tight junction‐related protein in lipopolysaccharide‐stimulated Caco‐2 cells. Nutrients, 11, 5 10.3390/nu11051001 PMC6567143 31052468

[fsn31481-bib-0024] Zhang, J. , Deng, B. , Jiang, X. , Cai, M. , Liu, N. , Zhang, S. , … Liu, S. (2019). All‐trans‐retinoic acid suppresses neointimal hyperplasia and inhibits vascular smooth muscle cell proliferation and migration via activation of AMPK signaling pathway. Frontiers in Pharmacology, 10, 485 10.3389/fphar.2019.00485 31143119 PMC6521230

